# Dispensing practices for anti-malarials in the La Nkwantanang-Madina municipality, Greater Accra, Ghana: a cross-sectional study

**DOI:** 10.1186/s12936-019-2897-5

**Published:** 2019-07-30

**Authors:** Charles Enyaah Amankwa, Harriet Affran Bonful, Kofi Agyabeng, Priscillia A. Nortey

**Affiliations:** 1 Department of Epidemiology and Disease Control, School of Public Health, University of Ghana, Legon, P.O Box LG 13, Accra, Ghana; 2 Ghana College of Pharmacists, Cantonments, P.O Box CT10740, Accra, Ghana; 3 Department of Biostatistics, School of Public Health, University of Ghana, Legon, P.O Box LG 13, Accra, Ghana

**Keywords:** Dispensing practice, Anti-malarials, Dispensers, Clients, Community pharmacies, Ghana

## Abstract

**Background:**

Despite recent strides made towards reducing the emergence of artemisinin resistance, inappropriate dispensing practices for anti-malarials in both private and public sectors affect treatment outcomes negatively. In Ghana, private retail pharmacies are the most accessible health facilities for managing diseases of common occurrence. However, there is growing concern about the number of patients harmed by dispensing errors in the management of malaria in retail pharmacies. Although considerable work has been done in this area, several questions regarding dispensing practices remain unanswered. This study, therefore, sought to investigate the predictors of appropriate dispensing practices for anti-malarials in community pharmacies in the La Nkwantanang-Madina municipality of Greater Accra, Ghana.

**Methods:**

A cross-sectional analytic study was conducted in sixty-one randomly selected community pharmacies in the La Nkwantanang-Madina. Data from 230 clients and 106 dispensers were analysed. It was checked for internal consistency and completeness then entered and analysed using STATA I/C version 14.0. Frequencies, Chi square tests, and logistic regression analyses were conducted, accounting for clustering.

**Results and discussion:**

Of the 106 dispensers interviewed, 71.4% were medicine counter assistants. The mean age of dispensers was 30.4 years (SD 8.8). Over 88.0% of clients were advised to complete the full course of their anti-malarials. However, the 8-h loading dose principle for artemether-lumefantrine was not explained to 88.3% of the clients. More than half of the clients (52.2%) were given appropriate dispensing information on anti-malarial use. Most clients (66.1%), were dispensed anti-malarials without malaria tests. Dispensers with more than a 10-years experience were less likely to dispense artemisinin-based combinations appropriately relative to dispensers with less than 2 years experience (AOR = 0.04, 95% CI 0.002–0.802 p-value = 0.036) while pharmacy interns were about 19 times more likely (AOR = 18.5, 95% CI 1.40–245.6 p-value = 0.03) to dispense artemisinin-based combinations appropriately compared to pharmacists.

**Conclusion:**

Dispensing practices for anti-malarials is unsatisfactory. There is a need to enforce existing legislation with educational programmes directed towards dispensers especially those with more than 10 years experience. Specific adherence to the World Health Organization Test, Treat and Track initiative should be encouraged to ensure effective use of anti-malarials.

## Background

The accurate use medicines play an important role in healthcare delivery. The provision of appropriate medicines can help alleviate symptoms, prevent recurrence of disease and restore patient’s health. Nonetheless, inappropriate use of medicines remains a major public health issue globally [1, 2].

In developing and low middle income countries, there has been considerable evidence of inappropriate drug use due to poor dispensing practices, inadequate package labelling and poor instruction given to clients on usage of medicines [3, 4].

In contrast to the developed regions, private retail pharmacies and Licensed Chemical Shops play an important role in promoting access to basic health services in sub-Saharan Africa. In Ghana, for example, private retail pharmacies and Licensed Chemical Shops, who are registered suppliers of specific over-the-counter medicines are the most accessible health facilities available for managing commonly occurring diseases [5]. Empirical studies have shown that the majority of families seek treatment for mild febrile diseases in retail pharmacies as compared to public health facilities [6, 7]. Their operations are regulated by the Pharmacy Council (PC) of Ghana under the Health Professions Regulatory Bodies Act 857 [8]. Community pharmacies are typically managed by pharmacists (individuals who hold a degree in pharmacy, completed the required internship programme and passed professional qualifying examination) and other pharmaceutical support staff; Medicine Counter Assistants (MCA—support staff trained to complement the role of pharmacy technicians, owing to acute shortage of the technicians) and pharmacy technicians.

Retail pharmacies are patronized primarily because they are readily accessible, have longer opening hours and clients may spend less time to be served compared to the public health facilities [5, 9, 10]. However, they are poorly monitored and may not be operating professionally [10–12]. In a study conducted by Buabeng in 2010, an alarming 77% of patients used incorrect dosage or did not complete the course of their anti-malarials [13]. The same study showed that (54%) of the patients who purchased from community pharmacies took their anti-malarials inappropriately possibly due to poor dispensing information [13]. Other researchers have rather observed that a greater proportion of patients had informed understanding of adverse effects compared to ‘how to use’ anti-malarials [11, 14]. These point to the importance of adequate dispensing and its pivotal role in preventing medication use errors [15, 16].

The dispensing process forms an integral part of the quality use of medicines and is the core professional role of a pharmacist. It ensures the safe and effective provision of medication to the general public. Dispensing involves all activities from the receipt of the prescription to the time the medicine is issued out to the patient [15]. The role of the pharmacist has traditionally been to provide patients with appropriate pharmaceutical care, to maintain the quality of pharmaceutical products and to ensure that patients take their medicines rationally [5, 8, 10, 11, 13].

In recent times, pharmacists’ role has evolved to advising physicians and other health professionals about drug therapy, its adequacy, side effects and possible interactions. In the community pharmacy setting, however, pharmacists and other support staff may not have the opportunity to interact with physicians and therefore, treat their clients empirically [13, 17]. This may have dire effects on patients once vital information is missed and often resources involved in patient care prior to dispensing may be wasted.

The introduction of rapid diagnostic testing (RDT) in the private sector in Ghana has made it easier for dispensers in community pharmacies to diagnose malaria definitively and treat confirmed malaria cases. However, in most malaria endemic areas, oral anti-malarials purchased from retail outlets are mainly obtained through client requests without laboratory confirmation [6, 18, 19]. Therapy initiated without conclusive test outcomes may not only accelerate artemisinin resistance but lead to the possible progression and aggravation of the actual cause of disease [17, 20–23].

Evidence from a cross-sectional survey and other studies point to a possible link between poor dispensing practices and its effect on anti-malarial resistance [24–26]. There is, therefore, a need for continuous assessment of the quality of dispensing of anti-malarials as this will inform policy makers and the National Malaria Control Programmme (NMCP) on areas to be targeted during the roll-out of interventions. Although considerable work has been done in this area, past studies on dispensing practices have primarily focused more on dispensing in public health facilities, understanding the role of pharmacists, assessing staff/patient knowledge, policy guidelines adherence among many others [13, 16, 23, 27–29].

However, there is limited information on dispensing practices among community pharmacies especially in the Greater-Accra region of Ghana. This study aims to address that gap by assessing factors associated with dispenser’s practices for anti-malarials in the La Nkwantanang-Madina municipality.

## Methods

The study was conducted in La Nkwantanang-Madina located in the Greater Accra Region, one of the ten administrative regions in Ghana at the time of the study. La Nkwantanang-Madina is located on the south-eastern part of the country along the Gulf of Guinea. It is estimated to have a population of 111,926 representing 2.8% of the region’s total population [30]. Pharmaceutical services in the district are delivered in private, public, quasi-governmental health centers and mission facilities. The provision of pharmaceutical care in the health delivery system in the municipality of La Nkwantanang-Madina is considered to be critical and is dominated by the private sector. The study population included dispensers at post in retail community pharmacies and their clients who had purchased oral anti-malarials for use and were exiting from the pharmacies. Pregnant women and individuals with cognitive disabilities who had purchased anti-malarials were excluded from the study.

This study employed an analytical cross-sectional approach among 61 selected community pharmacies in La Nkwantanang-Madina, from May to July 2017. A total of 122 retail community pharmacies and 36 wholesale outlets were obtained from Pharmacy Council, Ghana. This study employed a simple random sampling technique to select 61 community pharmacies, excluding wholesalers and licensed chemical shops. Data were gathered from three sources: dispensers, clients, and pharmacies.

Questionnaires were administered for data collection. The client and dispensers’ questionnaires were adapted from the WHO modified paper on good dispensing practices, as well as Training Manual for Licensed Chemical Sellers in Ghana [16]. Data obtained from respondents were collected, reviewed for completeness and entered using EpiData (Version 3.1). Double data entry was done to minimize errors and ensure the reliability of data collected.

The clients’ questionnaire captured data on age, gender, educational level, religion, marital status, National Health Insurance (NHI) registration status, rapid diagnostic test (RDT) check, client’s occupation, purchased drugs and dispensing information. Information on the number of times Pharmacy Council had visited the pharmacy within the last 6 months, availability of reference materials on Malaria Case Management (MCM) and availability of MCM wall chart were obtained from the dispensers in their respective pharmacies.

The client and dispenser data were merged and checked for internal consistency and completeness using simple summary statistics of the selected variables. Age of respondents (both dispensers and clients) were measured as completed years. Frequencies were used to describe the sociodemographic characteristics of dispensers and clients. Where there was evidence of skewness in the data, means were estimated for continuous variables after log transformation.

The dependent variable, dispensing practice was measured as a binary ordinal categorical variable (appropriate and inappropriate dispensing practices) (Table 1). This was assessed based on 13 key indicators for measuring dispensing practices (Table 4) [20, 27].

**Table 1:** Dispensing practice classification

Score (n = 13)	Dispensing practice
Less than 9	Inappropriate
At least 9	Appropriate

Chi square tests of associations were used to determine associations between the independent and independent categorical variables. Fisher’s exact tests were used when sample sizes were found to be small (< 5 per cell). Univariable and stepwise multivariable logistic regression analyses were performed to determine the predictors of dispensing practices at 95% confidence level and α-value of 0.05.

## Results

Overall, client sample size of 248 and 106 dispensers were obtained after data validation. However, there were 18 missing values in the client dataset. Therefore, 230 and 106 completed questionnaires were obtained for client and dispenser data, respectively.

Table 2 shows the characteristics of dispensers in community pharmacies at the time of the survey. Although hundred and twenty-four (124) dispensers initially consented to partake in the study, only a hundred and six (106) completed questionnaires were obtained in the 61 pharmacies visited. Females constituted 72%. The mean age of these dispensers was 30.4 (SD = 8.8) years ranging from 18 years to 70 years.

**Table 2:** Socio-demographic characteristics of dispensers in community retail pharmacies in La Nkwantanang-Madina-2017

Characteristics	Frequency (n = 106)	Percentage (%)
Sex		
Male	34	32.1
Female	72	67.9
Age (mean, sd)	(30.4, 8.8)	
Age group (years)		
15–24	21	19.8
25–34	55	51.9
35–44	22	20.8
> 45	8	7.6
Marital status		
Single	69	65.1
Married	37	34.9
Highest level of education		
None	–	–
Primary/elementary	1	0.9
Secondary	30	28.3
Tertiary	73	68.9
Vocational/technical	2	1.9
Religion		
Muslim	3	2.8
Christian	103	97.2
Ethnicity		
Ewe	19	17.9
Akan	54	50.9
Ga/Adangme	29	27.4
Northerner	4	3.8
Professional category	(n = 97)	
Pharmacist	14	14.4
M.C.A	71	73.2
Pharmacy technician	3	3.1
Pharmacist Intern	8	8.3
Other (manager/owner)	1	1.0

The characteristics of the study participants (clients) are presented in Table 3. More than half of the clients (60.9%) were females. The mean age was 33.70 (SD = 10.3) years and ranged from 15 years to 69 years. The majority (53.6%) were married. About thirty-nine percent (38.7%) had completed secondary school whereas primary and tertiary had nearly equal proportions of 25.0% and 25.4% respectively. Seventy-two (29.4%) clients were traders while 26.6% were artisans (Table 3).

**Table 3:** Socio-demographic characteristics of clients who patronize retail pharmacy services in the La Nkwantanang-Madina-2017

Characteristics	Frequency (n = 248)	Percentage (%)
Sex		
Male	96	38.7
Female	151	60.9
Age (mean, sd)	(33.7, 10.3)	–
Marital status		
Single	102	41.1
Married	133	53.6
Separated/divorced	5	2.0
Widow/widower	8	3.2
Highest level of education		
None	8	3.2
Primary/elementary	62	25.0
Secondary	96	38.7
Tertiary	63	25.4
Vocational/technical	19	7.7
Religion		
Muslim	35	14.1
Christian	211	85.1
Traditional	2	0.8
Ethnicity		
Ewe	64	25.8
Akan	100	40.3
Ga/Adangme	43	17.3
Northerner	36	14.5
Other	5	2.0
Occupation		
Trader	72	29.0
Artisan^a^	66	26.6
Professional^b^	34	13.7
Office worker^c^	27	10.9
Unemployed	11	4.4
Student	35	14.1
Other	3	12.0

^a^To a worker in a skilled trade especially one that involves making things by hand. Eg hairdressers, painters, etc.

^b^Includes skilled professionals such as nurses, teachers, etc.

^c^Mainly people who work in offices e.g. bankers

More than half (58%) of the clients were given accurate directions on the use of their ACT’s with 88.7% being advised to complete the full course. However, most dispensers (88.3%) did not explain the 8-h loading dose principle to their clients (Table 4).

**Table 4:** Distribution of areas of assessment during dispensing and post-dispensing advice

Areas of assessment (advise)	Frequency (n = 248)	Percentage (100%)
Advice on how to take anti-malarials		
Accurate direction	103	58.5
Inaccurate direction	145	41.5
Taking after meals		
After meals	237	95.6
Before meals	11	4.4
Completion of full course		
Advised	220	88.7
Not advised	28	11.3
Risk of side effects		
Advised	53	21.4
Not advised	195	78.6
Labelling		
Labelling in ink		
Yes	230	92.7
No	18	7.3
Clear instruction		
Clear	216	87.1
Not clear	32	12.9
Legibility to client		
Legible	223	89.9
Illegible	25	10.1
Label captured		
Quantity of drugs dispensed		
Yes	226	91.1
No	22	8.9
Frequency of administration		
Yes	219	88.3
No	29	11.7
Exact time to take the drug		
Yes	29	11.7
No	219	88.3
Duration of administration		
Yes	191	77.0
No	57	23.0

Assessment was from clients exit interviews

Chi square test for trends (Table 5) showed significant associations between each of the following variables: NHIS status (chi = 5.69, *p *< 0.05), dispenser’s marital status (chi = 6.55, *p* < 0.05), access to reference materials (chi = 9.59, *p* < 0.05), type of recommender (chi = 16.54, *p* < 0.05), awareness of guidelines (chi = 5.38, *p* < 0.05) and dispensing practices.

A crude analysis of the association between dispensing practices and the predictor variables showed that marital status, access to reference materials, dispenser’s awareness of guidelines, National Health Insurance Scheme registration status and number of supervisory visits within the last 6 months were associated with the outcome of dispensing practices. However, adjusting for all other variables, dispenser’s age, level of education, professional category and level of experience were significant predictors of dispensing practices (Tables 5, 6).

**Table 5:** Chi square test of association of socio-demographic characteristics of dispensers and dispensing practices

Variables	Dispensing practices				χ^2^	p-value
	Appropriate		Inappropriate			
	Frequency	%	Frequency	%		
Age group						
15–24	14	11.6	11	10.0	8.05	0.184
25–34	59	49.2	58	52.7		
35–44	29	24.2	36	32.7		
45+	18	15.0	5	4.6		
Total	121	100.0	110	100.0		
Sex	N = 120		N = 110			
Male	34	28.3	33	30.0	0.08	0.802
Female	86	71.7	77	70.0		
Educational level	N = 120		N = 110			
None	–	–	–	–	1.47	0.577
Primary	1	0.8	–	–		
Secondary	42	35.0	36	32.7		
Tertiary	76	63.3	72	65.5		
Vocational/technical	1	0.8	2	1.8		
NHIS status	N = 120		N = 110			
Have NHIS	59	49.2	37	33.6	5.69	0.048*
Not have NHIS	62	51.2	73	66.4		
Professional category	N = 120		N = 110			
Pharmacist	14	11.8	15	13.6	6.51	0.239
Medicine Counter Assistant	85	71.4	88	80.0		
Pharmacy Technician	6	5.0	2	1.8		
Pharmacy Intern	11	9.2	3	2.7		
Other	3	2.5	2	1.8		
Years of experience	N = 119		N = 110			
< 2 years	28	25.0	14	14.6	3.73	0.535
3–5 years	41	36.6	39	40.6		
6–9 years	31	27.7	29	30.2		
> 10 years	12	10.7	14	14.6		
Marital status	N = 112		N = 96			
Single	82	68.3	57	51.8	6.55	0.046*
Married	38 N = 120	31.7	53 N = 110	48.2		
Access to wall chart						
Yes	13	10.8	24	21.8	5.13	0.095
No	107	89.2	86	78.2		
Access to reference material	N = 120		N = 110			
Yes	53	44.2	71	64.5	9.59	0.02*
No	67	55.8	39	35.5		
Supervisory visits	N = 120		N = 110			
Once	48	40.0	58	52.7	10.71	0.127
Twice	40	33.3	20	18.2		
Three times	3	2.5	5	4.6		
More than 3	3	2.5	0	0		
Can’t remember	26	21.7	27	24.6		
Recommender	N = 120		N = 110			
Dispenser	67	55.8	63	57.3	16.54	0.024*
Relative	16	13.3	5	4.6		
Friend	2	1.7	0	0		
Self	16	13.3	30	27.3		
Media	0	0	2	1.8		
Unspecified	19	15.8	10	9.1		
Awareness of guidelines	N = 120		N = 110			
Yes	112	93.3	92	83.6	5.38	0.019*
No	8	6.7	18	16.4		
In-service training (last 12 months)	N = 120		N = 110			
Yes	24	20.0	36	32.7	4.86	0.259
No	70	58.3	53	48.2		
Can’t remember	26 N = 120	21.7	21 N = 110	19.1		

Statistically significant associations are marked with *

**Table 6:** Univariate and multivariate logistic regression estimates of the socio-demographic predictors of appropriate dispensing practices of dispensers

Characteristics	Crude OR (95% CI)	p-value	Adjusted OR (95% CI)	p-value
Age group				
15–24 (reference)	1		1	–
25–34	0.80 (0.24–2.72)	0.714	1.97 (0.31–12.61)	0.465
35–44	0.63 (0.17–2.32)	0.482	7.76 (0.89–67.37)	0.063
> 45	2.82 (0.53–15.10)	0.218	15.23 (25.91–5893.9)	0.001*
Sex				
Male (reference)	1			
Female	1.08 (0.57–2.07)	0.80	0.95 (0.35–2.64)	0.927
Educational level				
None (reference)	1		1	–
Primary	1 (omitted)	–	1 (omitted)	–
Secondary	7.83e−7	–	8.5e−7 (4.3e−8–1.7e−5)	0.001*
Tertiary	7.03e−7	–	9.2e−7 (5.4e−8–1.6e−5)	0.001*
Vocational/technical	3.36e−7	–	5.8e−6 (1.22e−7–3e−4)	0.001*
Professional category				
Pharmacist (reference)	1		1	–
MCA	1.03 (0.33–3.22)	0.952	1.64 (0.43–6.25)	0.461
Pharm. Technician	3.21 (0.42–24.71)	0.255	1.90 (0.39–9.33)	0.421
Pharm. Intern	3.93 (0.53–29.08)	0.176	18.5 (1.40–245.6)	0.03*
Other	1.61 (0.54–4.75)	0.383	4.91 (0.40–60.40)	0.208
Marital status				
Single	1	–	1	–
Married	0.49 (0.25–0.99)	0.047*	0.20 (0.040–1.01)	0.051
NHIS status				
Have NHIS (reference)	1		1	
Don’t have NHIS	0.52 (0.27–0.99)	0.050*	0.41 (0.12–1.07)	0.067
Access to wall chart				
Yes	1	–		
No	2.30 (0.85–6.23)	0.10	3.32 (0.85–13.00)	0.082
Experience				
< 2 years (reference)	1	–		
3–5 years	0.53 (0.19–1.49)	0.219	0.47 (0.09–2.36)	0.348
6–9 years	0.53 (0.15–1.92)	0.328	0.25 (0.026–2.50)	0.209
> 10 years	0.43 (0.10–1.75)	0.231	0.04 (0.002–0.802)	0.036*
Awareness of guidelines				
Aware (reference)	1			
Unaware	0.37 (0.15–0.86)	0.022*	0.45 (0.16–1.24)	0.122
Access to reference material				
Yes	1	–		
No	2.30 (1.14–4.63)	0.020*	2.10 (0.61–7.20)	0.240
Supervisory visits				
Once	1	–	1	
Twice	2.42 (1.15–5.06)	0.020*	1.35 (0.38–4.84)	0.636
Three times	0.73 (0.25–2.08)	0.543	0.51 (0.12–2.14)	0.345
> Three	1 (omitted)	–	1 (omitted)	–
Can’t remember	1.16 (0.55–2.50)	0.688	1 (omitted)	–
Recommender				
Dispenser	1	–	1	–
Relative	3.00 (1.18–7.68)	0.022*	7.40 (1.03–53.41)	0.047*
Friend	1 (omitted)	–	1 (omitted)	–
Self	0.50 (0.25–0.99)	0.046*	0.20 (0.07–0.53)	0.002*
Media	1 (omitted)	–	1 (omitted)	
Unspecified	1.79 (0.66–4.85)	0.248	3.22 (0.78–13.35)	0.104

Statistically significant associations are marked with *

*OR* odds ratio

**Fig. 1:**
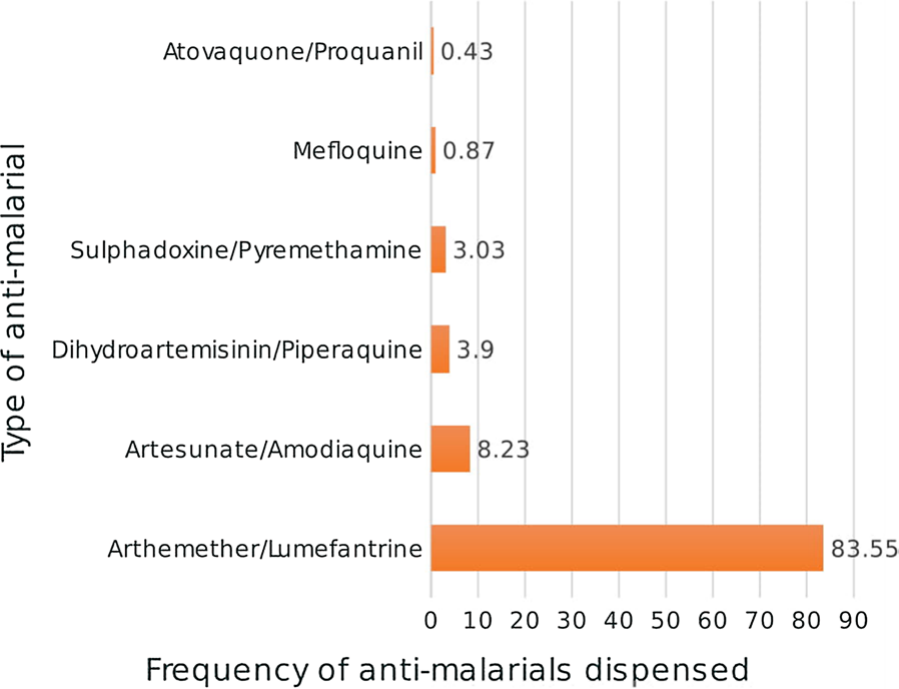
Bar chart showing the distribution of anti-malarials dispensed in community pharmacies within La-Nkwantanang Madina municipality, July 2017

Pharmacist interns were 18.5 times more likely to dispense anti-malarials appropriately compared to practicing pharmacists (AOR 18.50, 95% CI 1.40–245.6; *p* < 0.05). Also, relative to dispensers with less than 2 years working experience, dispensers with more than 10 years’ experience were less likely to dispense Artemisinin Combination Therapy (ACT’s) appropriately (AOR 0.04, 95% CI 0.002–0.802 *p* < 0.05). The most dispensed anti-malarial was artemether-lumefantrine (83.55), while atovaquone-proguanil was the least dispensed (Fig. 1).

## Discussion

The over-consumption of oral anti-malarials in the private drug retail sector is a major threat to the health of the people. With concerns of overdiagnosis of malaria, poor dispensing and subsequent increase in antimicrobial resistance, key strategic options such as the distribution of rapid diagnostic test kits, peer education and training of dispensers in the private retail sector was done to scale up malaria prevention and treatment [28, 31, 32].

This study showed that dispensing practices for anti-malarials is generally unsatisfactory at the study site. With about 48% of dispensers (47.8%) dispensing anti-malarials inappropriately, a high fraction of inappropriate dispensing practices was similarly observed by Alotaibi and Abdelkarim in a survey conducted in Dawadmi, Saudi Arabia [33]. Consumers perceived that pharmacists (48%) do not give enough counselling about their medications [33]. The observation could, however, be attributed to time constraints either from the service provider or the client [34]. In connection to this, some authors have indicated interruptions during dispensing; low staff strength, inadequate knowledge, lack of training and dispenser fatigue as likely causes of inappropriate dispensing practices [35]. This high rate of inappropriately dispensed ACT medicines and poor counselling if not addressed could impede progress in the fight against anti-malarial resistance in Ghana.

This study could not clearly establish an association between in-service training and dispensing practices. In contrast, several studies have associated a positive relationship between training and dispensing practices. In a study where, dispensing practice was measured as the ability to identify the recommended anti-malarial, the odds of knowing the recommended treatment was significantly higher amongst participants with health training [28].

Dispensers with more than 10 years’ experience were less likely to dispense ACT appropriately (AOR = 0.04, 95% CI 0.002–0.802 *p*-value < 0.05) while pharmacy interns were about 19 times more likely (AOR = 18.5, 95% CI 1.40–245.6 *p*-value < 0.05) to dispense ACT appropriately compared to pharmacists. Hussein and Ibrahim, in a similar study, reported that dispensers with less than one-year work experience had a better knowledge in Lahore, thus reflecting in their dispensing practices [4]. This might be linked to a greater number of graduate pharmacists with updated knowledge on the newer trends in managing diseases of common occurrence at the community level. Cordina et al. in their study asserted that younger pharmacists identified more with current trends in the practice of pharmacy relative to their colleagues [36].

**Fig. 2:**
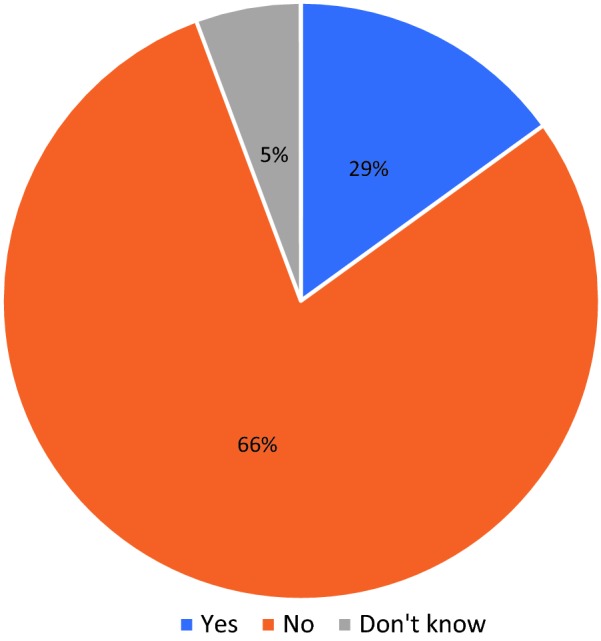
Pie chart showing the proportion of clients who were tested for malaria before being dispensed anti-malarials

The role of accurate diagnosis with the newly introduced rapid diagnostic test kits cannot be underestimated. Ansah et al., in a cluster randomized trial conducted among LCS in the erstwhile Dangme West District of Ghana, demonstrated that providing RDT’s for malaria in the private drug retail sector significantly reduced dispensing of anti-malarials to patients without malaria [37]. However, some patients are not tested prior to initiation of treatment for malaria. Findings from this study reports that an overwhelming majority (66%) of the patients were not tested before being dispensed oral anti-malarials (Fig. 2). This finding is consistent with a similar study in Tanzania which reported that about 98% of patients were treated presumptively for malaria without performing an RDT [19]. This phenomenon has been observed in the Ghanaian public health facilities as well, as other reports suggest that over 40% of patients were treated presumptively for malaria [37, 38].

Brugha and colleagues have pointed out that motivation for profit in the private health sector may compromise the quality of care and numerous cross-sectional studies have attributed this phenomenon to inadequate training services for community pharmacy attendants, lack of supervision by regulatory authorities and the lack of confidence in the diagnostic test kits [39]. This finding illustrates the potential risk of malaria misdiagnosis which may accelerate the burden of anti-malarial resistance as has been recorded in South Eastern Asia [24, 26, 40]. Further research is, therefore, required to get the views and perception of dispensers. Monitoring and evaluation of training interventions may provide useful insights on dispensing practices. In so doing, pharmacists can introduce methods and strategies to expand their contribution in the dispensing process.

### Study limitations

The study had some few limitations. Firstly, responses to dispensing practices and the last attendance to an in-service training may have been influenced by recall biases. Also, the cross-sectional nature of the study may limit the ability to infer causality.

The findings of this research may represent the reality in municipalities with comparable characteristics as the study site.

## Conclusion

Community pharmacy dispensing practices were found to be unsatisfactory. Almost half of the clients (47.8%) were given inappropriate dispensing information on the use of their anti-malarials. Dispensers with more than 10 years working experience were less likely to dispense ACT medicines appropriately, while pharmacy interns were more likely to dispense ACTs appropriately. In view of this, strategies to educate and update the knowledge base of all dispensers focusing on those with more than 10 years working experience may be helpful. Education should be channeled on adherence and compliance to World Health Organization’s Test, Treat and Track initiative, as well to promote the appropriate use of anti-malarials.

## Data Availability

The datasets obtained and/or analysed during the current study are not available publicly, however, it will be made available from the corresponding author on reasonable request.

## Abbreviations

ACT: artemisinin-based combination therapy
AL: artemether–lumefantrine
CI: confidence interval
NMCP: National Malaria Control Programme
RDT: rapid diagnostic test
LCS: Licensed Chemical Sellers
PC: Pharmacy Council
NHI: National Health Insurance
MCM: Malaria Case Management
MCA: Medicine Counter Assistants
AOR: adjusted odds ratio
WHO: World Health Organization
